# Zanubrutinib‐Induced Acneiform Rash in a Patient With Waldenstrom’s Macroglobulinemia

**DOI:** 10.1155/carm/9415119

**Published:** 2026-02-02

**Authors:** Silvia Robuffo, Corrado Zengarini, Claudio Agostinelli, Elena Sabattini, Michelangelo La Placa, Bianca Maria Piraccini, Alessandro Pileri

**Affiliations:** ^1^ Department of Medical and Surgical Sciences, University of Bologna, Bologna, 40138, Italy, unibo.it; ^2^ Dermatology Unit, IRCCS Azienda Ospedaliero-Universitaria di Bologna, Bologna, 40138, Italy; ^3^ Haematopathology Unit, IRCCS Azienda Ospedaliero-Universitaria di Bologna, Bologna, 40138, Italy

**Keywords:** Bruton’s Tyrosine Kinase inhibitors, dermatological toxicities, Waldenström’s macroglobulinemia, zanubrutinib

## Abstract

Waldenström’s macroglobulinemia is a rare lymphoproliferative disorder that can be treated with Bruton’s Tyrosine Kinase inhibitors (BTKi), including zanubrutinib. Although zanubrutinib is associated with fewer off‐target effects than first‐generation BTKi, dermatologic toxicities may still occur. We report the case of an 81 year‐old man with Waldenström’s macroglobulinemia who developed a Grade I acneiform rash shortly after initiating zanubrutinib. The eruption, characterised by folliculocentric papules and pustules on the face and trunk, resolved with topical azelaic acid and salicylic acid, as well as oral azithromycin. Histology showed a perivascular and periadnexal CD3^+^ T‐cell infiltrate without epidermotropism. A Naranjo score of 8 supported a probable drug reaction. This report highlights the need for awareness of cutaneous side effects associated with newer BTKi to ensure prompt diagnosis and optimal patient management.

## 1. Introduction

Waldenström’s macroglobulinemia (WM) is a rare indolent B‐cell lymphoproliferative disorder characterised by bone marrow infiltration with lymphoplasmacytic cells and the presence of circulating monoclonal immunoglobulin M (IgM) protein [1]. Its clinical presentation is heterogeneous, ranging from asymptomatic or smouldering forms to symptomatic disease with peripheral cytopenias, hepatosplenomegaly, lymphadenopathy or hyperviscosity syndrome. In symptomatic patients, treatment is indicated, and several therapeutic options are available, including chemotherapy, monoclonal antibodies, proteasome inhibitors, B‐cell lymphoma 2 (BCL2) inhibitors and Bruton’s Tyrosine Kinase inhibitors (BTKi) [1].

Zanubrutinib is a second‐generation, highly selective BTKi approved for use in treatment‐naïve or relapsed/refractory WM patients, particularly those unsuitable for chemoimmunotherapy. In the ASPEN Phase III trial, it showed a favourable safety profile with fewer off‐target effects and better health‐related quality of life outcomes compared to ibrutinib [2]. However, as its clinical use expands, new adverse events—particularly cutaneous toxicities—are beginning to emerge and warrant further investigation.

We present the case of an 81‐year‐old man with WM who developed a zanubrutinib‐induced acneiform eruption shortly after therapy initiation. To our knowledge, detailed descriptions of acneiform rashes induced by zanubrutinib are scarce in the literature, and this report contributes to the characterisation and management of such dermatologic toxicities.

## 2. Case Presentation

The patient was diagnosed with WM at age 69 but had never received prior chemotherapy. His past medical history included occult hepatitis B virus infection, congestive heart failure with pleural effusion and hypertension. He had been managed with a watch‐and‐wait approach for the last 11 years due to prolonged clinical stability and absence of treatment‐triggering symptoms. Still, in early 2024, progressive disease with laborator deterioration (including rising IgM cytopenias) led to the decision to start systemic therapy with zanubrutinib in April 2024, at a dosage of 160 mg orally twice daily.

**FIGURE 1:**
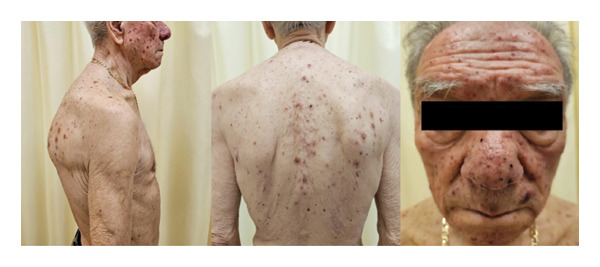
Clinical presentation of the acneiform eruption associated with zanubrutinib. Erythematous papules and pustules are visible on the face and upper trunk, predominantly involving seborrhoeic areas, consistent with Grade I CTCAE acneiform dermatitis.

**FIGURE 2:**
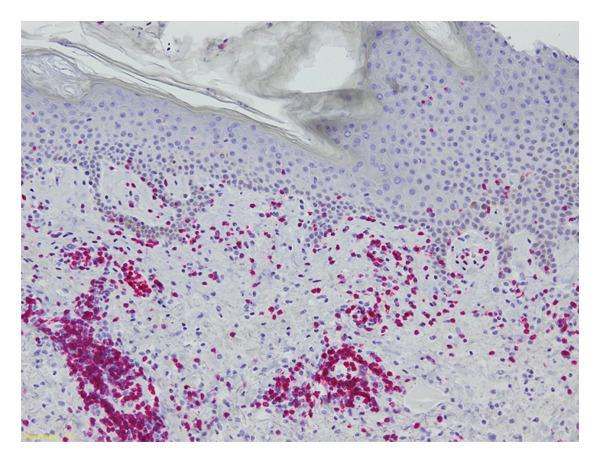
Histological examination of an acneiform lesion showing periadnexal and perivascular CD3^+^ T‐cell lymphoid infiltrate with preserved CD4/CD8 ratio. The lesion exhibits abundant macrophages, red blood cell extravasation and folliculocentric inflammation, without epidermotropism, consistent with a drug‐induced acneiform eruption (CD3 immunohistochemistry, 10 × magnification).

Three days after starting treatment, the patient developed an asymptomatic facial eruption that progressively extended to the upper trunk. A dermatological examination revealed erythematous papules and pustules centred on hair follicles, with occasional purpuric lesions distributed over seborrhoeic areas (Figure 1). A 6‐mm punch biopsy was performed, and histopathology revealed a periadnexal and perivascular CD3^+^ T‐cell lymphoid infiltrate with a preserved CD4/CD8 ratio, intact CD7 expression, numerous macrophages and extravasated red blood cells (Figure 2). No epidermotropism was observed. Bacterial and fungal cultures, as well as HSV1/2 PCR testing, were all negative. Based on the clinical and histological features, a diagnosis of Grade I acneiform eruption related to zanubrutinib was made. The adverse drug reaction was assessed as ‘probable’, with a Naranjo score of 8.

Treatment included a 3‐day weekly cycle of oral azithromycin for 4 weeks, along with daily application of salicylic and azelaic acid creams. The rash resolved entirely within 4 weeks, and zanubrutinib was continued without interruption.

## 3. Discussion

BTKi therapy is associated with a range of dermatologic adverse effects, which are believed to result from inhibition not only of BTK but also of off‐target kinases and from interference with epidermal growth factor (EGF) signalling [3]. Acneiform eruptions are a well‐recognised adverse effect of EGFR inhibitors and typically present within the first 2 weeks of treatment. These eruptions are characterised by folliculocentric papules and pustules, often lacking comedones, and predominantly involve seborrhoeic areas, such as the face, neck, retro auricular region, shoulders and upper trunk [4].

Although zanubrutinib is generally better tolerated than earlier‐generation BTKi, isolated cutaneous adverse events are beginning to be recognised. Wang et al. reported cases of papulopustular eruptions associated with zanubrutinib, but detailed clinical and histological characterisation remains limited [5]. To our knowledge, this is among the first reports to describe in detail a histologically confirmed acneiform eruption associated with zanubrutinib.

Recognition of this dermatologic adverse event is crucial, as proper diagnosis and conservative treatment can prevent unnecessary discontinuation of effective oncologic therapy. Further studies are needed to better define the incidence, risk factors and optimal management strategies for BTKi‐induced acneiform eruptions.

## Author Contributions

CRediT Author Statement: Silvia Robuffo: data curation, formal analysis and writing–original draft; Corrado Zengarini: conceptualisation, methodology, supervision and writing–review and editing; Claudio Agostinelli: investigation and data curation; Elena Sabattini: histopathological analysis and validation; Michelangelo La Placa: supervision and methodology; Bianca Maria Piraccini: supervision and resources; Alessandro Pileri: conceptualisation, project administration and supervision.

## Funding

This study was supported by University of Bologna. Open access publishing facilitated by Universita degli Studi di Bologna, as part of the Wiley ‐ CRUI‐CARE agreement.

## Ethics Statement

The authors have nothing to report.

## Consent

The patient in this manuscript has given written informed consent to publish their case details.

## Conflicts of Interest

The authors declare no conflicts of interest.

## Data Availability

Data are available on request from the authors.
